# Stigma-directed services (Stig2Health) to improve ‘linkage to care’ for people living with HIV in rural Tanzania: study protocol for a nested pre-post implementation study within the Kilombero and Ulanga Antiretroviral Cohort

**DOI:** 10.12688/aasopenres.13353.2

**Published:** 2022-08-05

**Authors:** Raphael Magnolini, Elizabeth Senkoro, Aneth Vedastus Kalinjuma, Olivia Kitau, Bernard Kivuma, Leila Samson, Anna Eichenberger, Getrud Joseph Mollel, Eileen Krinke, James Okuma, Robert Ndege, Tracy Glass, Herry Mapesi, Fiona Vanobberghen, Manuel Battegay, Maja Weisser

**Affiliations:** 1 Division of Infectious Diseases and Hospital Epidemiology, University Hospital of Basel, Basel, Switzerland; 2 Ifakara Health Institute, Ifakara, Tanzania; 3 St. Francis Referral Hospital, Ifakara, Tanzania; 4 School of Public Health, Faculty of Health Sciences, University of the Witwatersrand, Johannesburg, South Africa; 5 Department of Infectious Diseases, Inselspital, University Hospital Bern, Bern, Switzerland; 6 Swiss Tropical and Public Health Institute, Basel, Switzerland; 7 University of Basel, Basel, Switzerland; 8 University Psychiatric Clinics Basel, Basel, Switzerland; 9 University of Zurich, Zurich, Switzerland

**Keywords:** HIV/AIDS, Stigma, Linkage to care, HIV care, Sub-Sahara Africa, Study protocol

## Abstract

**Background: **HIV-related stigma is a major barrier to the timely linkage and retention of patients in HIV care in sub-Saharan Africa, where most people living with HIV/AIDS reside. In this implementation study we aim to evaluate the effect of stigma-directed services on linkage to care and other health outcomes in newly diagnosed HIV-positive patients.

**Methods**: In a nested project of the Kilombero and Ulanga Antiretroviral Cohort in rural Tanzania, we conduct a prospective observational pre-post study to assess the impact of a bundle of stigma-directed services for newly diagnosed HIV positive patients. Stigma-directed services, delivered by a lay person living with HIV, are i) post-test counseling, ii) post-test video-assisted teaching, iii) group support therapy and group health education, and iv) mobile health. Patients receiving stigma services (enrolled from 1 ^st^ February 2020 to 31 ^st^ August 2021) are compared to a historical control receiving the standard of care (enrolled from 1 ^st^ July 2017 to 1 ^st^ February 2019). The primary outcome is ‘linkage to care’. Secondary endpoints are retention in care, viral suppression, death and clinical failure at 6-12 months (up to 31 ^st^ August 2022). Self-reported stigma and depression are assessed using the Berger Stigma scale and the PHQ-9 questionnaire, respectively. The sample size calculation was based on cohort data from 2018. Assuming a pre-intervention cohort of 511 newly diagnosed adults of whom 346 (68%) were in care and on antiretroviral treatment (ART) at 2 months, a 10% increase in linkage (from 70 to 80%), a two-sided type I error rate of 5%, and 90% power, 321 adults are required for the post-implementation group.

**Discussion: **We expect that integration of stigma-directed services leads to an increase of proportions of patients in care and on ART. The findings will provide guidance on how to integrate stigma-directed services into routine care in rural sub-Saharan Africa.

## Introduction

The rollout of antiretroviral treatment (ART) for people living with HIV/AIDS (PLWHA) is one of the largest and most successful health interventions – especially in sub-Saharan Africa, where most affected persons live. Nevertheless, HIV programs aiming to reach the updated Joint United Nations Programme on HIV and AIDS (UNAIDS) goals of “95-95-95”, i.e. 95% of PLWHA diagnosed, 95% of people who are diagnosed receiving ART, and 95% of those receiving ART being virally suppressed, by 2025 are jeopardized by the loss of patients on all levels of the HIV care continuum – HIV testing, linkage to HIV care with ART initiation and retention in care for sustained viral suppression ^1– 3^. The highest loss of patients has been observed during ‘linkage to care’ – commonly defined as ‘patient entry into specialist HIV care after diagnosis’ ^2, 4– 8^. Poor care linkage appears to be particularly pronounced in sub-Saharan Africa ^2, 9, 10^.

An important reason for non-linkage is HIV-related stigma and discrimination ^11, 12^. HIV-related stigma is a complex concept, comprising of three important stigma mechanisms described for HIV infected individuals, i.e., enacted stigma (level to which PLWHA believe they have actually experienced prejudice and discrimination from others), anticipated stigma (the level to which PLWHA expect that they will experience prejudice and discrimination in the future), and internalized stigma (“felt” stigma or “self-stigmatization”, used to describe the way PLWHA feel about themselves) ^11, 13, 14^. PLWHA who perceive high levels of HIV-related stigma are 2.4 times more likely to delay enrolment in care compared to those who don’t ^15^. HIV-related stigma is associated with non- or involuntary HIV-status disclosure and denial of the HIV-status ^16^. Consequences are lower access to health care services, poor health-protective behaviors ^17^, delayed initiation of ART and low adherence to ART ^16, 18, 19^ resulting in poor treatment outcomes ^20– 22^. Protective factors against stigma are family cohesiveness, and social and emotional support ^23^. Depression and stigma are mutually associated, and both affect HIV treatment outcome ^20, 21, 24, 25^.

Despite the vast literature on HIV-related stigma and its negative impact on outcomes for PLWHA ^11^, few intervention studies have targeted the reduction of HIV-related stigma among PLWHA in different socio-cultural settings ^11, 26^. Different promising and cost-effective intervention strategies to improve care and health outcomes in resource-limited settings include task shifting from physicians to lay persons with lower-level qualifications ^27– 29^, video-assisted teachings (VAT), mobile Health (mHealth) ^30– 34^, group support therapy and group health education ^27, 35^. Advantages of VAT are the low cost, standardized delivery, and time-savings for healthcare staff due to its customized content and flexible dissemination channels ^23, 36^.

In Sub-Saharan Africa, mental health services addressing depression have shown to be a major treatment gap with lack of services in up to 90% ^25, 37– 39^ – mostly due to lack of trained healthcare professionals ^25^ or lack of accessibility and sustainability. Some data suggests that implementing task shifting in resource-limited settings can improve mental disorders among PLWHA by delegating care from psychotherapists to lay health care workers ^27, 28^. With mental health services still being very poorly available and not integrated into HIV care in sub-Saharan Africa, little is known about task shifting in this field.

The effectiveness and feasibility of combined interventional strategies has previously been evaluated ^3, 40, 41^, but to our knowledge the combined effect of stigma-directed services has not been studied.

## Protocol

### Study aim

The aim of this implementational study is to evaluate the effect of the impact of a bundle of stigma-directed services on linkage to care and other health outcomes for patients that are newly diagnosed with HIV. Stigma-directed services, delivered by a lay person living with HIV, are i) post-test counseling, ii) post-test video-assisted teaching, iii) group support therapy and group health education, and iv) mHealth. Stigma-directed services are aimed to reduce all levels of stigma within PLWHA, i.e., enacted stigma, anticipated stigma, and internalized stigma. Importantly, only the complete bundle of stigma-directed services to the outcomes can be assessed, the value of each individual service to the outcomes cannot be differentiated. We hypothesize that integration of combined stigma-directed services into routine care for patients newly diagnosed with HIV will increase timely linkage to care, retention in care and viral suppression. Additionally, we hypothesize patients’ self-reported stigma and depression within the first year will decrease.

### Study setting

The Chronic Disease Clinic Ifakara (CDCI) was established in 2004 as the governmental care and treatment center for PLWHA of the St. Francis Referral Hospital (SFRH) in Ifakara, South-western rural Tanzania. The clinic provides free HIV testing, care and antiretroviral drugs for PLWHA according to the National AIDS Control Program (NACP) guidelines. All patients are offered participation in a prospective patient cohort – the Kilombero and Ulanga Antiretroviral Cohort (KIULARCO) and are enrolled the day of diagnosis upon consent. KIULARCO is a collaborative project of the SFRH with the Ifakara Health Institute, the Swiss Tropical and Public Health Institute and the University Hospital Basel to allow research on needs and treatment outcomes of patients ^42, 43^.

### Study design

**Figure 1.**
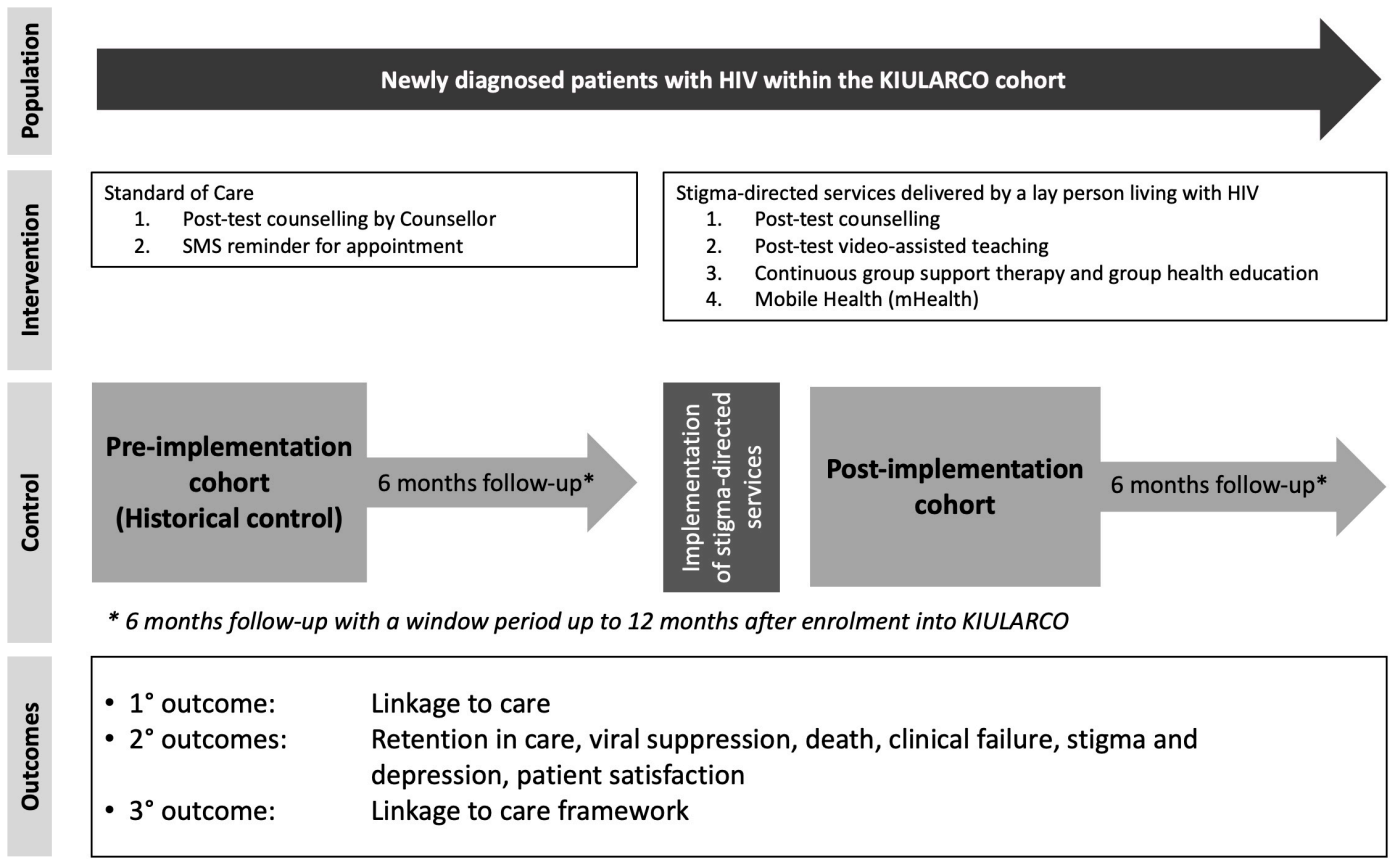
Study design of the stigma-directed prospective observational pre-post study.

We will conduct a prospective observational pre-post study nested within KIULARCO. All newly diagnosed patients with HIV consenting to KIULARCO will be offered stigma-directed services at enrolment from 1 ^st^ February 2020 onwards in addition to standard of care. Prior to Stig2health, the standard of care involved pre- and post-ART counselling in newly diagnosed patients as per national guidelines and clinical follow-up reminders by automated text messages to all patients attending the clinic with a registered phone number ( Figure 1).

### Recruitment, screening eligibility criteria

From 1 ^st^ February 2020 onwards, all newly HIV-diagnosed patients enrolled in KIULARCO and fulfilling eligibility criteria ( Table 1) will be offered stigma-related services by a professional counselor (VCT) on the same day.

**Table 1.** Eligibility criteria to receive stigma services during the post-implementational phase.

Inclusion criteria	Exclusion criteria
• Adults (≥15 years of age) • Newly diagnosed with HIV at the CDCI at the SFRH • Written informed consent to KIULARCO	• Current hospitalization for any reason • Indeterminate HIV test result • Non-consenting for KIULARCO • Too sick to answer the questions

CDCI = Chronic Disease Clinic Ifakara; SFRH = St. Francis Referral Hospital; KIULARCO = Kilombero and Ulanga Antiretroviral Cohort

### Informed consent for the Stig2Health nested study within KIULARCO

Stig2health will be nested within the KIULARCO and is part of the KIULARCO protocol, which has received initial ethical approval from Ifakara Health Institute institutional review board (IHI/IRB/No 16-2006) and National Institute for Medical Research (NIMR) (NIMR/HQ/R.8a/Vol.IX/620). Clearance for stigma interventions with updated consent and documentation were added to the KIULARCO protocol and approved by ethical committees within a respective amendment (IHI/IRB/AMM/No:08-2020 and IHI/IRB/AMM/No: 2-2021; NIMR/HQ/R.8c/Vol.I/764 and NIMR/HQ/R.8c/Vol.I/896). Patients will sign an informed consent form (see *Extended data* ^44^). Patients can either accept or reject stigma services. Patients who do not wish to receive them, receive standard of care clinical workup and treatment. Lay counselors will obtain oral consent from patients before asking stigma- and depression specific questions.

### Stigma services delivered by a lay person living with HIV

Well-trained lay counselors openly living with HIV (one male, one female) will be responsible for the conduction of all stigma-related services. Lay counsellors will be trained by different medical professionals and professional counselors over a duration of 4 weeks using a standard operating procedure aligned to the national guidelines (competency list in *Extended data*, Table S2 ^44^). The lay counselors will be selected from patients at our clinic and have either been living with HIV since birth or for multiple years. They will be trained in all aspects of the stigma services, including data entry and handling. Strategies to ensure program fidelity include standardized materials, structured education training, ongoing supervision, and training a sufficient number of lay counselors.

***1) Post-test counseling***

On the day of diagnosis, standardized post-test counseling with patients will be conducted by the lay counselor immediately after a newly confirmed positive HIV test and the first information is given by the professional counselor. The stigma-directed counselling will covers social support/disclosure, emotions/fears, basic knowledge of HIV transmission and steps towards positive health and living positively with HIV.

***2) Post-test VAT***

Thereafter, the lay counselor will show the patient a 16-minute video on a portable device (Samsung Tablet A, 10.1 inch) containing experiences of HIV-positive persons and information from healthcare workers from our clinic. While others have used video in similar situations ^45, 46^ our goal was to produce a video reflecting and capturing the local socio-cultural context and the hospital itself to increase relatability and recognition value in patients. The video is in Swahili language and use is restricted to the clinical purpose.

The recorded patients are PLWHA from Ifakara and health care workers from the CDCI who agreed to participate to develop a video to be shown to newly diagnosed clients. The study investigator obtained written informed consent from all video participants before the shooting took place. The investigators explained to each participant the purpose of the video.

In the video PLWHA report on how HIV and HIV-related stigma have affected their lives and how they integrated HIV into their daily lives. They talk about living positively with HIV, challenges they experienced, experience with disclosure, social support, and ART adherence. The video also contains information provided by healthcare professionals on HIV/AIDS such as disease manifestations, transmission, and treatment. Increased knowledge aims at reduction of fear and internalized stigma.

So far, no studies have examined content or other aspects of such an educational video, despite these having been used in similar situations ^23, 36^.

***3) Group support therapy and group health education***

Daily peer group support therapy and group health education will serve to discuss questions around HIV-related stigma and increase knowledge about HIV/AIDS for all interested patients. Group support therapy and group health education are based on participant-centered learning, social support ^47^, strength-based non-judgmental communication ^48^, identifying communalities ^49^, creating hope ^50^ and active listening ^51^. Culturally adapted learning materials and implementation tools will be used. Each session will consist of an introduction including group therapy rules, an opportunity to share problems ^50, 52^ and combines peer group support therapy with group health education ^27^. We will use active learning techniques like role play, brainstorming, discussions with active listening ^51^, storytelling and exercises with picture cards. Five sessions, comprising of a duration of 30-45min and maximum participant number of 10 patients per session, with different standardized content for group support therapy, including integration of HIV and ART in everyday life, internalized and community stigma, depression and coping as well as disclosure and relationship, have been elaborated upon (Table S1, *Extended data* ^44^). The positive effects of implementation of group support therapy and group health education ^27, 35^ within similar settings has been described previously.

***4) Mobile Health (mHealth)***

We will be using mHealth to ensure early care engagement during the first weeks after diagnosis. In addition to appointment reminders delivered by automated text messages (SMS) to all patients attending the clinic as standard of care at the CDCI, the lay counselor will call newly diagnosed patients 3 days before their first and second clinical follow-up after diagnosis (two-way communication voice call). To minimize fear of involuntary HIV disclosure to people overhearing the conversation, the health message only encourages participants to care for their health with an HIV neutral content and not mentioning the health care facility or personal patient information ^53^. Mobile phone calls to remind patients about their clinic visits has successfully been integrated into HIV care ^34, 40, 41, 54^ and support the patient to feel valued by the clinic with a wide acceptability ^53, 54^.

### Depression, stigma and patient satisfaction questionnaires

Stigma- and depression-related questionnaires will be completed together with the patient one month after start of ART, to assess stigma and create awareness. The questions will also be a starting point to discuss those topics with the lay counselors. Both questionnaires, for depression and stigma, are versions of adapted scales previously translated into Kiswahili and validated in Tanzania ^55– 57^ and have been successfully used in sub-Saharan Africa ^58– 61^. Signs of depression are measured using the Patient Health Questionnaire-9 (PHQ-9) ^56, 57^, which analyzes symptoms of depression on a 4-point Likert-type scale ^58, 59^. An adapted version of the Berger’s stigma scale ^62^ will be used to measure internalized and perceived stigma within PLWHA on a 7-items questionnaire rated on a 5-point Likert-type scale ^55^. Any patient in whom major depression (depression score of ≥ 9) or suicidal ideation is detected will be evaluated by the clinician and if major depressive disorder is confirmed they will be managed according to Tanzanian guidelines and the ICD10 diagnosis is recorded in the patient system. In an end-of-study questionnaire at 6 months, we will additionally ask for patient satisfaction and acceptance of each service by using the “Net Promoter Score” (NPS), a 0-to-10 numerical customer satisfaction metric ^61, 63^. The net promoter score was effectively adapted for use in the medical field to measure patient experience, also within sexual and reproductive health clinics ^61, 64^.

### Outcomes

The primary endpoint in this study will be ‘linkage to care’. Despite ‘linkage to care’ being a key HIV indicator for public health monitoring, multiple definitions used in different studies make comparisons challenging ^10^. For Stig2healh ‘linkage to care’ will be defined as “being in active care and on ART at 2 months”. The time period was chosen as there might be patients with postponed ART start due to co-infection with tuberculosis (Tb) or cryptococcal meningitis. All secondary and tertiary outcomes can be found in Table 2. Tertiary endpoints were added at a later stage of the study protocol development according to recent research done by our study team on the ‘linkage to care framework’ ^10^.

**Table 2.** Secondary and tertiary endpoints.

Endpoint	Timepoint after enrolment into KIULARCO	Definition
**Secondary endpoints**		
Pre- and post-implementation cohort		
Retention in care	at 6 months *	“patients known to be alive and receiving ART at the end of the follow-up period of 6 months ^65”^.
Viral load suppression	at 6 months	“having a viral load of less than 400 copies of HIV RNA per milliliter“ ^66, 67^
Death	until 6 months	
Clinical failure	until 6 months	“occurrence of any new WHO AIDS- defining disease, death or loss to follow- up at any visits” ^68– 70^
Proportion of patients with self- reported depression comparing pre-/post cohorts	at 6 months	Categorical scores and continuous scores
Level of self-reported stigma comparing pre-/post cohorts	at 6 months	Continuous scores
Post-implementation cohort only		
Proportion and changes of patients with self-reported depression within the post cohort	at 1 month **, 6 months	Categorical scores and continuous scores
Level and changes of self-reported stigma within the post cohort	at 1 month, 6 months	Continuous scores
Patient satisfaction of each individual stigma service	at 6 months	Categorical scores and continuous scores
**Tertiary endpoints**		
Proportion of participants with:		
i) a first laboratory evaluation	at 3 months ***	Having a laboratory result form with sampling date within the given time period following enrolment.
ii) a first clinical evaluation	at 3 months	Having a clinical visit with date within the given time period following enrolment.
iii) with ART initiation	at 3 months	Being initiated on ART within the given time period following enrolment.
iv) a clinical follow-up visit following ART initiation	at 3 months	Having a clinical visit with date after ART initiation within the given time period following enrolment.

*6 months with a time window from 6 up to 12 months, **1 month with a time window from 2–6 weeks, ***3 months without a time window; ART= Antiretroviral therapy; RNA= ribonucleic acid, HIV= human immunodeficiency virus; AIDS= acquired immunodeficiency syndrome; KIULARCO = Kilombero and Ulanga Antiretroviral Cohort

### Participant timeline

The lay counseling and video intervention will be offered the day of diagnosis and enrollment into KIULARCO. On patient request it can be moved to day 1. During routine follow-up visits scheduled according to the National AIDS control program at month 1, 2, 3, 6, 9 and 12, group support therapies will be offered. The mHealth phone call will be conducted 3 days prior to the first two clinical follow-up visits. Depression and stigma questionnaires for the post-implementation cohort will be filled during the first month (2–6 weeks) and at the first viral load measurement visit that is usually conducted at 6 months after enrolment into KIULARCO, with an additional assessment of patient satisfaction of stigma services at the same time point. As the 6-month time point may be missed in some occasions, a window period up to 12 months after enrolment into KIULARCO will be accepted. End of study will be 31 ^st^ August 2022. Stigma and depression questionnaires for the pre-implementation cohort will be assessed in patients at their 6 month follow-up visit if they were not subjected to any stigma intervention. Timing of stigma specific patient visits and data collection can be found in Table 3.

**Table 3.** Timing of Stig2Health related patient visits and data collection within routine follow-up visit scheduling according the National AIDS control program.

Study period	Baseline visit	Follow-up visits	
**Time point** (Window period)	**Day 1** **(day 0–1)**	**Month 1** **(week 2–6)**	**Month 6** **(week 26-52)**
Patient Information and Informed Consent of KIULARCO (registration)	X		
Post-test counselling (professional counsellor)	X		
**Stigma** baseline services - Lay Counselling - Video Intervention	X X		
**Stigma** mHealth service (phone calls done prior to clinical visits)		X	
**Stigma** Invitation to group therapy sessions and group health education		X	X
Vital signs (nurse)	X	X	X
Visit by study doctor - History - Physical Examination - Drug prescription	X	X	X
Laboratory testing (venipuncture) - Blood: Full blood Count - Liver enzymes and Creatinine - Cryptococcus, tuberculosis, hepatitis, syphilis according KIULARCO protocol - Viral load - CD4 cell count	X X X X		X X X X
Self-reported adherence/Pill box return		X	X
PHQ-9 depression questionnaire		X	X
Adapted Berger stigma questionnaire		X	X
Net Promoter Score			X

*First follow-up visit at 6 months where the first viral load is assessed, with a window period up to 12 months will be allowed, KIULARCO = Kilombero and Ulanga Antiretroviral Cohort, CD4 = Cluster of differentiation 4; PHQ-9 = patient health questionnaire-9

### Sample size

For the primary endpoint – linkage to care – we assumed an increase from 70 to 80% based on a systematic review on strategies to improve linkage to care rates in different urban areas in sub-Saharan Africa and a linkage to care rate of 68% within KIULARCO in 2018 ^71^. The sample size calculation was based on cohort data from 2018, when 511 adults were newly diagnosed and of whom 346 (68%) were in care and on ART at 2 months. Assuming a pre-intervention cohort of 511 adults, a 10% increase in linkage (from 70 to 80%), a two-sided type I error rate of 5%, and 90% power, 321 adults are required for the post-intervention group. The full sample size calculation can be found in the *Extended data* ^44^. Based on the enrolment into KIULARCO from 2017 of >500 patients, we expect to reach the required sample size within one year.

### Data collection and management

Numbers from routine testing activities captured in books will be transferred to an excel sheet saved on a local password-protected server. Data from patients enrolled in KIULARCO will be entered into an electronic medical record system (openMRS). Documentation of stigma services will be done using a counselling form within openMRS and will be filled by the clinician and the professional counselor. In addition, stigma and depression questionnaires will be captured in Open Data Kit (ODK) by the lay counselor with final scores entered into openMRS.

Data from the stigma and depression questionnaires will be extracted weekly, cleaned and stored on a study computer and in a data sharing platform (Alfresco, hosted by Swiss TPH). The routinely collected KIUARCO data will be extracted from the IHI sever and processed for research use quarterly. The data extraction for this study will be done at the end of study data collection and will be stored in a secured cloud system.

### Confidentiality

Participant data will be anonymized and accessible only to authorized study personnel. Anonymity of the participants will be guaranteed when presenting the data at scientific meetings or publishing them in scientific journals. All medical information obtained within this study will be considered confidential.

## Statistical methods

Participant characteristics at enrolment into KIULARCO will be described using descriptive statistics for both primary outcome and secondary outcomes. The association between the primary outcome and the intervention bundle will be assessed using the logistic regression model and the measure of effect will be estimated in terms of adjusted odds ratio and thereafter risk differences (with 95% confidence interval) will be estimated ^72, 73^. The variable selection procedure for adjusted models will be explained in a statistical analysis plan.

The linkage to care framework will be constructed based on four stages defined within three months since being enrolled into KIULARCO. Steps to be considered include (a) laboratory assessment; (b) clinical evaluation; (c) ART initiation; and (d) clinical follow-up after treatment initiation. The multinomial linear regression model will be used to assess the association between linkage stages and intervention bundle. The risk difference will be estimated using the approach described in the primary outcome.

The association between binary secondary outcomes such as retention in care, viral load, and self-reported-depression and intervention bundle will be analyzed using logistic regression models. Self-reported stigma and depression scores will be analyzed using linear regression models. Further, within the post-intervention group, the change in self-reported stigma and depression will be analyzed using linear regression models.

To assess patient satisfaction with individual services ^61, 64^ in the post-cohort, the proportion of detractors, passive, and promoters will be estimated for each intervention and the final net promoter score will be calculated by deducting detractors from promoters.

There are two potential sources of missing data which include loss to follow-up and transfer to other HIV clinics. Transfers will affect primary and secondary outcomes. Lost to follow-up will be an additional source of missing data in all secondary outcomes. In case the percentage of the missing data will exceed 10% of all recruited participants, missing data techniques will be used to address the bias due to omission. Details will be provided in a separate full statistical analysis plan. Data management and analyses will be done using Stata software version 16 and SAS version 9.4 (SAS Institute Inc., Cary, NC, USA).

## Discussion

With a four-component bundle of stigma-directed services we expect to increase ‘linkage to care’ by 10% from 70 to 80% post-implementation. We expect to gain knowledge on the value of stigma-directed services and to observe an increase in linkage to care proportions, virological suppression rates and retention in care within the first year. By increasing linkage to care and retention in care, the HIV-cascade in a rural setting in sub-Saharan Africa should improve. We expect to further understand the complex social, cultural, and intrapersonal construct of stigma and to get to know more about the fears and concerns of patients, on how to target them and to be able to encourage patients to take steps towards a positive health behavior. HIV-directed stigma and discrimination are still crucial barriers that affect accessibility and acceptability of HIV healthcare services. With this study we hope to provide guidance on how to integrate simple, low-cost, and sustainable stigma-related services into routine care in rural sub-Saharan Africa, but also raise awareness on HIV-related stigma and health outcomes in this setting and worldwide. The results of this prospective stigma-directed study may provide more insight into task shifting to and collaborations with lay health care workers in primary health care facilities in low-resource settings and has the potential to lead to policy changes regarding integration of stigma and mental health-directed services in sub-Saharan Africa.

### Study status

Follow-up period until August 31 ^st^, 2022

### Dissemination plan

Dissemination will occur through publication in a peer-reviewed journal. Additional dissemination will occur through presentations at conferences nationally and internationally and by informing district health authorities.

## Data availability

### Underlying data

No underlying data are associated with this article

### Extended data

Zenodo: Extended data for Stig2Health implementational study: Stigma-directed services to improve 'linkage to care' for people living with HIV in rural Tanzania. https://doi.org/10.5281/zenodo.5916625 ^44^

This project contains the following extended data:

- KIULARCO_ICTenglish_2020_11_15.pdf (KIULARCO Cohort Informed Consent form in English)
- KIULARCO_ICTswahili_2020_11_15.pdf (KIULARCO Cohort Informed Consent form in Swahili)
- Stig2Health Adapted Berger Stigma Scale.pdf
- Stig2Health End of study questionnaire.pdf
- Stig2Health Information for Sample size calculation.pdf
- Stig2Health PHQ-9 Depression questionnaire swahili.pdf
- Stig2Health Table S1- Outline group support therapy and group health education.pdf
- Stig2Health Table S2 Overview lay counselor competency list.pdf

Data are available under the terms of the Creative Commons Attribution 4.0 International license (CC-BY 4.0).

Permission to publish the adapted Berger stigma scale and PHQ-9 depression questionnaire in Swahili was received from Prof. Sylvia Kaaya on 28.01.2022.

The post-test video is not available publicly, because consent for publication was not obtained and the publication could pose a threat to confidentiality of participants. It was agreed that this video is only available for research and clinical use and not for public viewing to ensure confidentiality of participants. Inquiries on the video are coordinated through the University Hospital Basel. Requests will be handled by Prof. Dr. med. Maja Weisser m.weisser@unibas.ch

## KIULARCO study group

Aschola Asantiel, Farida Bani, Manuel Battegay, Theonestina Byakuzana, Adolphina Chale, Francisca Chuwa, Ivana di Salvo, Gideon Francis, Hansjakob Furrer, Tracy Glass, Yvone Haridas, Speciosa Hwaya, Aneth V Kalinjuma, Joshua Kapunga, Bryson Kasuga, Andrew Katende, Namvua Kimera, Yassin Kisunga, Olivia Kitau, Bernard Kivuma, Thomas Klimkait, Ezekiel Luoga, Herry Mapesi, Slyakus Mlembe, Mengi Mkulila, Margareth Mkusa, Dorcas K Mnzava, Getrud J Mollel, Lilian Moshi, Germana Mossad, Dolores Mpundunga, Athumani Mtandanguo, Selerine Myeya, Sanula Nahota, Robert C. Ndege, Reginna Ndaki, Suzan Ngahyoma, Agatha Ngulukila, Jacopo Nicoletti, Alex John Ntamatungiro, Amina Nyuri, James Okuma, Daniel H Paris, Jacqueline Nkouabi, Aloyce Sambuta, Leila Samson, Elizabeth Senkoro, Giorge Sigalla, Jenifa Tarimo, Juerg Utzinger, Fiona Vanobberghen, Maja Weisser, John Wigay, Herieth Wilson.
