# Supplementary material for: Stigma-directed services (Stig2Health) to improve ‘linkage to care’ for people living with HIV in rural Tanzania: study protocol for a nested pre-post implementation study within the Kilombero and Ulanga Antiretroviral Cohort
Source: AAS Open Res. 2022 Aug 5;5:14. Originally published 2022 Mar 21. [Version 2] doi: 10.12688/aasopenres.13353.2 (PMC9648364; doi:10.12688/aasopenres.13353.2)
Supplement: Peer review history

# Peer review: Reviewer response for version 1

**DOI:** 10.21956/aasopenres.14499.r29267

**Published:** 2022-07-22

**Authors:** Richelle Harklerode

**Affiliations:** 1 Institute for Global Health Sciences, University of California, San Francisco, San Francisco, CA, USA

The manuscript describes a study protocol for the Stig2Health project to improve linkage to HIV care services. The article is well written and will contribute to understanding if HIV-related stigma services can improve the care cascade in Tanzania. The following are points of potential revision:

1. Noted as a low-cost method, for sustainability please consider including the cost of the training and daily cost of lay counselors.
2. There are a number of types of stigma, the questionnaire seems to focus on stigma from those informed of patient’s HIV status, it would be useful to state this within the article.
3. For the group counseling to be able to replicate the study, I recommend providing additional information of length of the five sessions, frequency/time between sessions, and tracking outcomes based on the number of sessions patients participated in.
4. The depression questionnaire only has the Swahili version uploaded. If the patient is found to be depressed what treatment is provided? It would be useful to note if the services are different for those with depression and for those without depression.
5. There is a shift in tense between sections of the manuscript, I recommend revision to be consistent.
6. As the study is in progress, it is unlikely to change the end of study questionnaire, although it seems fitting to ask if they found the services helpful, instead of asking if they would recommend to other people living with HIV, as this could be confusing since that is not the patient’s role.

Is the study design appropriate for the research question?

Yes

Is the rationale for, and objectives of, the study clearly described?

Yes

Are sufficient details of the methods provided to allow replication by others?

Partly

Are the datasets clearly presented in a useable and accessible format?

Not applicable

Reviewer Expertise:

HIV, HIV care cascade, disease surveillance, monitoring and evaluation

I confirm that I have read this submission and believe that I have an appropriate level of expertise to confirm that it is of an acceptable scientific standard.

# Response

**Published:** 2022-07-29

**Authors:** Raphael Magnolini

**Affiliations:** University Hospital Basel, Switzerland

1. We will include the costs of lay counselors in the main manuscript.
2. Amended, the article now states: “An adapted version of the Berger’s stigma scale will be used to measure internalized and perceived stigma within PLWHA”.
3. Amended.
4. The depression questionnaire (PHQ-9) is a translation of a publicly available questionnaire in English language. The next steps if depression was detected was amended in the text, although that falls outside of the scope of this research.
5. Amended, all in future tense.
6. We thank the reviewer for this recommendation, although as stated the study is ongoing and this cannot be incorporated at this stage. We included this question as established tool (NPS questionnaire).

# Peer review: Reviewer response for version 1

**DOI:** 10.21956/aasopenres.14499.r29251

**Published:** 2022-06-27

**Authors:** Graeme Hoddinott

**Affiliations:** 1 Desmond Tutu TB Centre, Department of Paediatrics and Child Health, Faculty of Medicine and Health Sciences, Stellenbosch University, Stellenbosch, South Africa

The manuscript describes a study protocol for the Stig2Health project. Overall, it is written clearly and all necessary information included. Three points of further clarification / revision may be useful:

1. The tense shifts between past, future, and present tense throughout. I suggest that as a protocol manuscript, this should be future tense throughout - perhaps confused because the project may in fact already have started?
2. Stangl *et al.* ^1^ provide an overall conceptual framework for HIV stigma. Different domains of stigma (e.g., enacted, anticipated, and internal) have different impacts on outcomes (e.g., care seeking) and are intervened on differently (e.g., post test counselling addresses anticipated and internal stigma domains, but not enacted). It may be useful to include a section on your conceptual framework of stigma and how these intersect with each intervention component.
3. It is unclear that each of the intervention components are of equal value to the outcome, or how these interface with each other. E.g., do they work as a package or does each address a different aspect of stigma that combined addresses the outcome? Again, it may be useful to further clarify why these intervention components were selected in combination.

Congratulations again to the co-authors and good luck with implementing the project.

Is the study design appropriate for the research question?

Yes

Is the rationale for, and objectives of, the study clearly described?

Yes

Are sufficient details of the methods provided to allow replication by others?

Yes

Are the datasets clearly presented in a useable and accessible format?

Not applicable

Reviewer Expertise:

Tuberculosis, HIV, HIV stigma, mixed data evaluations.

I confirm that I have read this submission and believe that I have an appropriate level of expertise to confirm that it is of an acceptable scientific standard.

## References

[1] : The Health Stigma and Discrimination Framework: a global, crosscutting framework to inform research, intervention development, and policy on health-related stigmas. *BMC Medicine* .2019;17(1) : 10.1186/s12916-019-1271-3 10.1186/s12916-019-1271-3 30764826PMC6376797

# Response

**Published:** 2022-07-29

**Authors:** Raphael Magnolini

**Affiliations:** University Hospital Basel, Switzerland

1. Amended, all in future tense.
2. Amended, we further elaborated on the different mechanisms of stigma.
3. Amended, only the full bundle of stigma-directed intervention to the outcomes can be assessed, individual services cannot be differentiated.
